# Research on the optimization model of anti-breast cancer candidate drugs based on machine learning

**DOI:** 10.3389/fgene.2025.1523015

**Published:** 2025-04-10

**Authors:** Zhou Dong, Hong Chen, Yuchen Yang, Hairong Hao

**Affiliations:** School of Information Engineering, Xi’an Eurasia University, Xi’an, China

**Keywords:** breast cancer, machine Learning, quantitative structure-activity relationship models(QSAR), particle swarm optimization(PSO), ADMET Properties, drug screening, biological Activity

## Abstract

Breast cancer is one of the most common malignancies among women globally, with its incidence rate continuously increasing, posing a serious threat to women’s health. Although current treatments, such as drugs targeting estrogen receptor alpha (ERα), have extended patient survival, issues such as drug resistance and severe side effects remain widespread. This study proposes a machine learning-based optimization model for anti-breast cancer candidate drugs, aimed at enhancing biological activity and optimizing ADMET (Absorption, Distribution, Metabolism, Excretion, Toxicity) properties through multi-objective optimization. Initially, grey relational analysis and Spearman correlation analysis were performed on the molecular descriptors of 1,974 compounds, identifying 91 key descriptors. A Random Forest model combined with Shapley Additive Explanations (SHAP) values was then used to further select the top 20 descriptors with the greatest impact on biological activity. The constructed Quantitative Structure-Activity Relationship (QSAR) model, using algorithms such as LightGBM, Random Forest, and XGBoost, achieved an R^2^ value of 0.743 for biological activity prediction, demonstrating strong predictive performance. Additionally, a multi-model fusion strategy and Particle Swarm Optimization (PSO) algorithm were employed to optimize both biological activity and ADMET properties, thereby improving the prediction of Caco-2, CYP3A4, hERG, HOB, and MN properties. For example, the best model for predicting Caco-2 achieved an F1 score of 0.8905, while the model for predicting CYP3A4 reached an F1 score of 0.9733. This multi-objective optimization model provides a novel and efficient tool for drug development, offering significant improvements in both biological activity and pharmacokinetic properties, with practical implications for the optimization of future anti-breast cancer drugs.

## 1 Introduction

Over two million women are diagnosed with breast cancer each year, and some of these patients progress to advanced stages, urgently requiring effective treatments (Sung et al., 2021; Waks and Winer, 2019). While existing treatment options have extended survival, issues such as drug resistance and side effects persist (Giaquinto et al., 2022; Lumachi et al., 2011), creating a pressing need for the development of new anti-breast cancer drugs, particularly those targeting estrogen receptor alpha (ERα) and optimizing ADMET (Absorption, Distribution, Metabolism, Excretion, Toxicity) properties (Marra et al., 2020). With the rapid advancements in computer science and technology, machine learning has provided new solutions for drug design and optimization (Mak et al., 2023; Zhavoronkov et al., 2019). By constructing Quantitative Structure-Activity Relationship (QSAR) models based on compound structural features and biological activity data (Cherkasov et al., 2014), and integrating various machine learning algorithms, it is possible to efficiently predict the biological activity and ADMET properties of new compounds, reducing the time and cost of drug development (Jimé et al., 2020). Furthermore, optimization algorithms such as Particle Swarm Optimization (PSO) have shown excellent performance in multi-objective optimization tasks (Liu et al., 2021; Liu et al., 2024; Poli et al., 2007), enhancing both the biological activity and ADMET properties of compounds, thus providing powerful tools for drug screening and optimization.

Based on this background, the present study proposes a machine learning-based optimization model for anti-breast cancer candidate drugs. By integrating QSAR models, multi-model fusion techniques, and the PSO algorithm, this study aims to achieve multi-objective optimization of anti-breast cancer compounds, enhancing their biological activity against ERα while ensuring excellent ADMET properties. Here is the experimental procedure in this paper:

Phase 1: Data preprocessing, where 225 features with all zero values are removed and the data is normalized. A gray relational analysis is performed to select the 200 molecular descriptors most related to biological activity, followed by Spearman coefficient analysis, retaining 91 features. Then, Random Forest combined with SHAP value analysis is used to select the top 20 molecular descriptors with the most significant impact on biological activity (Table 2).

Phase 2: Using pIC50 (negative logarithm of the IC50 value) as the target variable, 10 regression models are used to predict the 20 selected features. By comparing evaluations, LightGBM, RandomForest, and XGBoost are identified as the best performers. To further improve prediction accuracy, these three models are combined using three ensemble methods: simple averaging, weighted averaging, and stacking. Finally, the stacking ensemble model is used to predict the pIC50 values for 50 target compounds and calculate their corresponding pIC50 (half-maximal inhibitory concentration) values, with the final results recorded in “ERα_activity_test.csv.”

Phase 3: After removing the 225 features with all zero values in Phase 1, Random Forest is used for recursive feature elimination (RFE) on the remaining 504 features. This selects 25 important features for each of the five ADMET (Absorption, Distribution, Metabolism, Excretion, and Toxicity) properties: Caco-2, CYP3A4, hERG, HOB, and MN. Using these selected features, 11 machine learning classification models are constructed. By comparing evaluation metrics such as F1 score and ROC curve, the best models for predicting Caco-2, CYP3A4, and hERG are identified as LightGBM, XGBoost, and NaiveBayes, respectively, with XGBoost being the best model for predicting MN. Finally, use the selected models to predict the classification results for 50 target compounds on Caco-2, CYP3A4, hERG, HOB, and MN, with the final results recorded in “ADMET_test.csv.”

Phase 4: First, a single-objective optimization model is constructed to improve the inhibition of ERα (Estrogen Receptor Alpha) biological activity while satisfying at least three ADMET properties. A total of 106 feature variables with high correlation to biological activity and ADMET properties from Phases 2 and 3 are selected. Regression and classification models are constructed based on these features to create the single-objective optimization model. Finally, a Particle Swarm Optimization (PSO) algorithm is used for multi-objective optimization search. Through multiple iterations, the best solution from each iteration is recorded and gradually converges to obtain the optimal value range. The final results are recorded in “results.csv.”

## 2 Related work

Breast cancer is one of the most common malignant tumors among women worldwide. Although current treatments such as surgery, radiotherapy, chemotherapy, and endocrine therapy have extended patient survival, these methods still have limitations due to the heterogeneity, drug resistance, and severe side effects associated with breast cancer (Hong and Xu, 2022; Belachew and Sewasew, 2021). Endocrine therapies targeting estrogen receptor alpha (ERα), such as tamoxifen and letrozole, have played a key role in treating ERα-positive breast cancer. However, as treatment progresses, these therapies increasingly face drug resistance, limiting their clinical application (Marra et al., 2020). Additionally, these drugs are associated with side effects such as cardiotoxicity and hepatotoxicity, creating an urgent need to develop new candidate drugs that not only address biological activity but also optimize ADMET (Absorption, Distribution, Metabolism, Excretion, Toxicity) properties (Caron and Nohria, 2018; Larroquette et al., 1986; Xu et al., 2015).

Recent advances in computer science and artificial intelligence have opened new avenues for drug design and optimization, offering substantial potential for overcoming these limitations (Rodrigues and Schneider, 2022; Stokes et al., 2020b; Schneider et al., 2020). Specifically, machine learning (Zitnik et al., 2019; Vamathevan et al., 2019) has proven to be a powerful tool for predicting the biological activity and ADMET properties of novel compounds, leveraging vast amounts of molecular descriptors and biological activity data (Mak et al., 2023; Zhavoronkov et al., 2019; Stokes et al., 2020a; Jimé et al., 2021). Traditional Quantitative Structure-Activity Relationship (QSAR) models, which correlate the physicochemical properties of compounds with their biological activity, have long been the cornerstone of drug development (Cherkasov et al., 2014; Jimé et al., 2020; Xu et al., 2015). However, these models often struggle to handle the complex nonlinear relationships between molecular features, limiting their ability to provide accurate predictions (Chen et al., 2020). To address this, recent studies have increasingly relied on multi-model fusion techniques, which combine the advantages of multiple models to improve prediction accuracy and stability (Lin et al., 2022; Chen and Guestrin, 2016). For instance, gradient boosting models such as LightGBM and XGBoost are particularly adept at handling high-dimensional data and capturing complex nonlinear relationships, making them widely used in predicting biological activity and ADMET properties (Shou, 2020; Lei et al., 2016).

The success of drug development depends not only on the biological activity of the drug but also on its ADMET properties. Favorable ADMET properties are crucial for the successful conversion of a candidate compound into an effective drug (Er-rajy et al., 2022; Ahmad et al., 2023). Some studies have utilized machine learning algorithms for classification and regression predictions of ADMET properties, achieving significant success in predicting permeability, metabolism, toxicity, and other pharmacokinetic attributes (Atallah et al., 2013; Komura et al., 2023). Algorithms such as Support Vector Machines (SVM), Random Forest, and XGBoost have been effective in screening compounds with favorable ADMET properties, reducing experimental costs and minimizing the risk of failure (Ferreira and Andricopulo, 2019; Huang et al., 2021).

However, optimizing multiple objectives simultaneously, such as enhancing biological activity and improving ADMET properties, remains a significant challenge in drug development (Luukkonen et al., 2023a). Traditional optimization methods struggle to effectively manage the trade-offs between these competing objectives (Deb et al., 2002). Particle Swarm Optimization (PSO), a swarm intelligence optimization technique that simulates cooperative search behavior within a population, has become a powerful tool for multi-objective optimization tasks, including drug design (Poli et al., 2007; Luukkonen et al., 2023b; Wang et al., 2018). PSO has been effectively applied to simultaneously optimize biological activity and ADMET properties, achieving the global optimal selection of candidate drugs and balancing these key attributes (Merk et al., 2018).

Building on these advances, this study integrates machine learning models with optimization algorithms such as PSO to successfully achieve multi-objective drug design. For example, integrating PSO with QSAR models has successfully enabled multi-objective optimization of both biological activity and ADMET properties in drug design. Additionally, multi-model fusion strategies have been employed to further improve predictive performance, combining different machine learning algorithms to reduce the bias of individual models and enhance overall prediction accuracy. These efforts have significantly advanced the development of drug optimization methods and tools.Based on previous work, this study proposes a novel machine learning-based optimization model for anti-breast cancer drugs. By combining QSAR models, multi-model fusion techniques, and the PSO algorithm, this study aims to simultaneously optimize the biological activity and ADMET properties of candidate compounds. Specifically, it enhances biological activity against ERα while ensuring optimal ADMET performance. This method not only provides an efficient and reliable tool for the development of anti-breast cancer drugs but also lays the foundation for future drug optimization research.

## 3 Dataset description

### 3.1 Dataset source

The core dataset used in this study is the “Anti-Breast Cancer Candidate Drug Optimization Modeling (2021)” dataset provided by the China Association for Science and Technology. This dataset is primarily focused on the biological activity prediction and ADMET property analysis targeting the breast cancer marker ERα, providing key data support for the machine learning modeling conducted in this study.

### 3.2 Dataset description

#### 3.2.1 ERα activity dataset (ERα_activity.xlsx)

Training Set (training table): Contains biological activity data for 1,974 compounds.

SMILES Format: The first column records the SMILES (Simplified Molecular Input Line Entry System) representation of each compound, which describes its structure.
IC50 Values: The second column lists the biological activity values against the ERα target in nanomoles (nM). Lower IC50 values indicate higher biological activity.
pIC50 Values: The third column records the negative logarithm of the IC50 values (pIC50), facilitating a more intuitive representation of the compounds’ biological activity; higher pIC50 values indicate stronger biological activity.

Test Set (test table): Contains the SMILES representation for 50 compounds, used for model prediction testing.

#### 3.2.2 Molecular descriptor dataset (Molecular_Descriptor.xlsx)

Training Set (training table): Includes 729 molecular descriptors for 1,974 compounds, describing each compound’s structure and its physicochemical properties.

SMILES Format: The first column contains the SMILES representation of the compounds, consistent with those in the ERα_activity.xlsx.
Molecular Descriptors: The subsequent 729 columns cover various molecular descriptors for each compound, including molecular weight, number of hydrogen bond donors, and hydrophobicity parameters (such as LogP), detailing their physicochemical characteristics and topological structure.

Test Set (test table): Contains the molecular descriptors for 50 compounds, used for model testing and evaluation.

#### 3.2.3 ADMET properties dataset (ADMET.xlsx)

Training Set (training table): Includes data on five ADMET properties for 1,974 compounds, all represented in a binary format.

Caco-2: Indicates the intestinal epithelial cell permeability of the compounds; 1 for good permeability, 0 for poor permeability.
CYP3A4: Indicates whether the compound can be metabolized by CYP3A4; 1 for metabolizable, 0 for non-metabolizable.
hERG: Indicates whether the compound has cardiotoxicity; 1 for toxic, 0 for non-toxic.
HOB: Indicates the oral bioavailability of the compound; 1 for good bioavailability, 0 for poor.
MN: Indicates whether the compound has mutagenicity; 1 for toxic, 0 for non-toxic.

Test Set (test table): Contains the SMILES representation for 50 compounds, used for model prediction and validation.

## 4 Experimental method and the solution results

### 4.1 Experimental design

This research consists of four main experimental steps, designated for selecting important molecular descriptors, predicting the biological activity of compounds, classifying ADMET properties, and multi-objective optimization.

#### 4.1.1 Feature selection and preprocessing

1. Feature Cleaning: Remove 225 molecular descriptors where all observations are zero to avoid redundancy and reduce the risk of overfitting.
2. Feature Normalization: Perform min-max normalization on the remaining 504 molecular descriptors to ensure that features are trained on the same scale, avoiding issues related to different dimensions affecting model training.
3. Grey Relational Analysis (GRA): Evaluate the correlation between pIC50 values and molecular descriptors using grey relational analysis, selecting the top 200 descriptors most relevant to biological activity.
4. Spearman Correlation Analysis: To further reduce feature redundancy, Spearman correlation analysis is used to process highly correlated features, retaining 91 key features to enhance model efficiency and accuracy.
5. Random Forest and SHAP Values: Further select 20 features with the greatest impact on biological activity.

#### 4.1.2 Construction of biological activity prediction model

1. Regression Model Selection: We utilize ten common machine learning regression models, including Linear Regression, Ridge, Lasso, ElasticNet, RandomForest, LightGBM, XGBoost, Gradient Boosting Decision Tree (GBDT), SVM, and Decision Tree.
2. Multi-Model Fusion: To improve the predictive performance of the model, we experimented with three fusion strategies on the three best-performing models (LightGBM, RandomForest, and XGBoost), including simple averaging, weighted averaging, and stacking. The stacking fusion showed the best effect.
3. Prediction Results: Use the best model to predict the pIC50 values for 50 test set compounds and convert them to IC50 values.

#### 4.1.3 Classification prediction of ADMET properties

1. Recursive Feature Elimination (RFE): Using RandomForest as the base model, the RFE method is applied to select features for ADMET properties, selecting 25 most representative molecular descriptors for each ADMET attribute.
2. Classification Model Selection: Utilize 11 classification models, including Logistic Regression, Naive Bayes, LDA, Decision Tree, RandomForest, AdaBoost, GradientBoosting, SVM, MLP, XGBoost, and LightGBM, to predict the ADMET properties of compounds.
3. Classification Effectiveness Assessment: Evaluate model performance using metrics such as the F1 score and ROC curve, and select the best models. The best classification models for different ADMET properties are LightGBM (Caco-2), XGBoost (CYP3A4 and hERG), NaiveBayes (HOB), and XGBoost (MN).
4. ADMET Property Prediction: Use the selected best models to predict the ADMET properties of 50 compounds.

#### 4.1.4 Multi-objective optimization

1. Single-Objective Optimization: Establish a single-objective optimization model aiming to enhance the biological activity (pIC50 value) of compounds while ensuring that at least three ADMET properties perform well.
2. Particle Swarm Optimization (PSO): Apply the PSO algorithm for global optimization of 106 important features, recording the optimal solution in each iteration, and ultimately finding the value range that provides the best performance in both biological activity and ADMET properties.
3. Final Results: Apply the optimized compound features to 50 test compounds, outputting their optimal predictive values.

### 4.2 Selection of molecular descriptors

#### 4.2.1 Data preprocessing and feature selection

##### 4.2.1.1 Data preprocessing

Basic statistical analysis is performed on the data provided in the “Molecular_Descriptor.xlsx” file. Some of the statistical results are shown in Table 1.

**TABLE 1:** Statistical information for selected molecular descriptors.

	nAtom	nHeavyAtom	nH	nB	nC
Count	1974	1974	1974	1974	1974
Mean	50.76	28.11	22.65	0	22.61
Std	18.09	8.07	10.78	0	6.63
Min	21	14	5	0	7
25%	36.25	21	14	0	17
50%	50	28	22	0	22
75%	62	34	29	0	28
Max	343	163	180	0	95

As observed, the values for the molecular descriptor nB are all zeros. Although a value of zero can have practical significance, prediction models are unable to recognize its meaning. Consequently, these variables are considered redundant features, which can affect the accuracy of the model. Therefore, we choose to remove these features, totaling the elimination of 225 molecular descriptors.

To eliminate the impact of dimensions and reduce the range of variables, the remaining features are normalized. The normalization formula is shown in Equation 1.

$$x=\frac{x_{i}-x_{\min}}{x_{\max}-x_{\min}}$$ (1)

Where x is the result after normalization, $x_{i}$ is the value in the original data table, $x_{\max}$ is the maximum value of a certain molecular descriptor in the original data table, and $x_{\min}$ is the minimum value of that molecular descriptor in the original data table.

##### 4.2.1.2 Grey relational analysis (GRA)

Grey relational analysis is used to identify the primary and secondary factors among the many influencing the development of a system. The fundamental idea is based on the degree of similarity in the geometric shapes of the sequence curves to determine the closeness of their relationships. The closer the curves are, the greater the degree of association between the corresponding sequences, and *vice versa*.Consider the reference sequence (biological activity) as $X_{0}$ and the compared sequences (influencing factors) as $\left(X_{1},X_{2},\cdot \;\cdot \;\cdot ,X_{m}\right)$ . The steps for calculating the grey relational analysis are as follows:

1. Calculate the correlation coefficients between each parameter in the compared sequences and the corresponding parameters in the reference sequence. Define the grey relational coefficients, which represents the extent of association between biological activity and each influencing factor, as presented in Equation 2.

$$\xi \left(x_{0}\left(k\right),x_{i}\left(k\right)\right)=\frac{a+\alpha b}{\left|x_{0}\left(k\right)-x_{i}\left(k\right)\right|+\alpha b}\;\left(i=1,2,\cdots ,m,k=1,2,\cdots ,n\right)$$ (2)

Where a is the minimum difference between the extremes, b is the maximum difference between the extremes, and α is the resolution coefficient (typically set to 0.5).

$$a=\min_{i}\underset{k}{\;\min}\left|x_{0}\left(k-x_{i}\left(k\right)\right)\right|$$

$$b=\max_{i}\underset{k}{\;\max}\left|x_{0}\left(k-x_{i}\left(k\right)\right)\right|$$

2. Calculate the grey relational degree. Define $r\left(X_{0},X_{i}\right)$ as the grey relational degree, obtained by calculating the mean of each column in the correlation coefficient matrix As shown in Equation 3.

$$r\left(X_{0},X_{i}\right)=\frac{1}{n}\sum_{k=1}^{n}\xi \left(x_{0}\left(k\right),x_{i}\left(k\right)\right)$$ (3)

**FIGURE 1:**
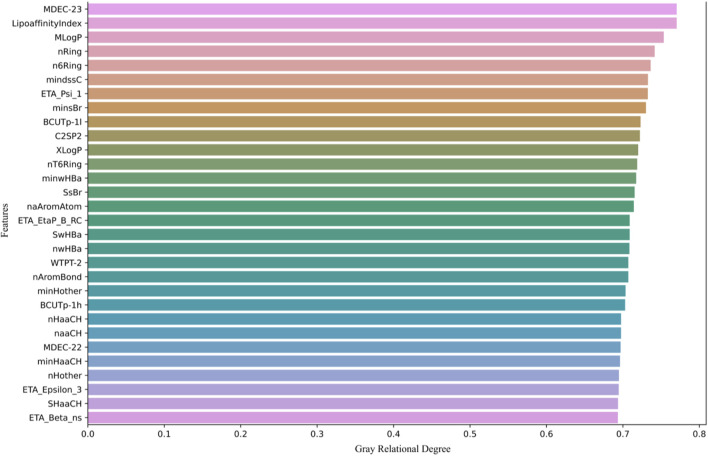
The top 30 molecular descriptors with the highest grey relational degree.

Next, we calculate the grey relational degree between each molecular descriptor and biological activity, retaining the top 200 molecular descriptors with the highest association values. As shown in Figure 1, only the top 30 molecular descriptors with the highest association values are displayed.

Figure 1 shows the top 30 molecular descriptors most strongly correlated with biological activity, selected through GRA. These molecular descriptors are ranked based on their grey relational degree with the pIC50 values (biological activity prediction values). The higher the grey relational degree, the stronger the correlation between the molecular descriptor and biological activity.

The molecular descriptors are sorted in descending order of grey relational degree, starting from the top. Each row represents a molecular descriptor, with the horizontal axis indicating its grey relational degree, ranging from 0 to 0.8. Descriptors such as MDEC-23, LipoaffinityIndex, MLogP, and nRing are displayed, all of which are used in subsequent models to predict molecular activity.

##### 4.2.1.3 Analysis of correlations between influencing factors

The Pearson correlation coefficient assumes that data follows a normal distribution and can only analyze linear relationships between variables. However, there are also complex nonlinear relationships between the data variables obtained. Therefore, the Spearman coefficient is chosen for analysis, as shown in Equation 4

$$\rho_{xy}=\frac{\sum_{i}\left(x_{i}-\bar{x}\right)\left(y_{i}-\bar{y}\right)}{\sqrt{\sum_{i}{\left(x_{i}-\bar{x}\right)}^{2}\sum_{i}{\left(y_{i}-\bar{y}\right)}^{2}}}$$ (4)

**FIGURE 2:**
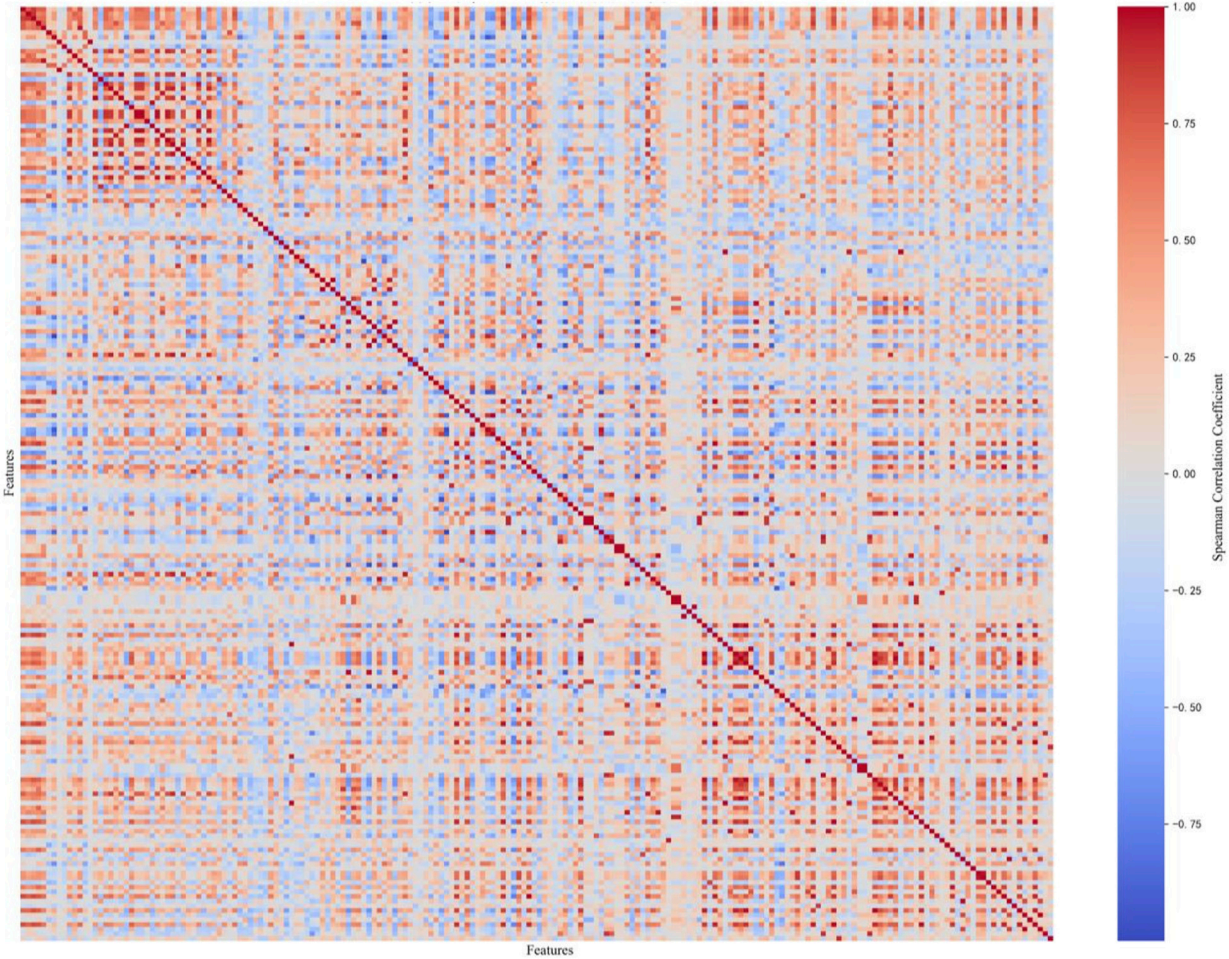
Spearman correlation coefficient heatmap between features.

Where x and y are the values of the two variables being analyzed, $\bar{x}$ and $\bar{y}$ are the mean values of the two variables. The Spearman correlation coefficient measures the monotonic relationship between two variables, with values ranging from −1 to +1. Positive values indicate a positive correlation between the variables, negative values indicate a negative correlation, and values close to 0 indicate a weaker correlation. By calculating the Spearman coefficients between the 200 molecular descriptors, we obtained the heatmap shown in Figure 2.

Figure 2 displays a heatmap of the Spearman correlation coefficient matrix for all molecular descriptors. In the heatmap, the intensity of the colors represents the magnitude of the Spearman correlation coefficient. Dark red indicates a strong positive correlation, dark blue indicates a strong negative correlation, and lighter colors represent weaker correlations. Highly correlated variables (greater than 0.85) were then filtered out, removing 109 molecular descriptors, and ultimately leaving 91 molecular descriptors.

##### 4.2.1.4 Variable selection model based on random forest

Subsequently, we used the remaining 91 molecular descriptors as feature variables to establish a random forest model for regression prediction of molecular activity and calculated the SHAP values for each molecular descriptor.

The random forest is an ensemble learning method used for tasks such as classification and regression. It builds multiple decision trees during the training process and uses the majority vote (for classification) or the average (for regression) of these trees’ predicted classes or values for final prediction. The random forest algorithm utilizes bagging (Bootstrap Aggregating) to create multiple training subsets from the original dataset.

Suppose the original dataset is $D=\{\left(x_{i},y_{i}\right){\}}_{i=1}^{n}$ , containing n samples. The bagging process generates B bootstrap samples $D_{b}$ , where $b\in \left\{1,2,\ldots ,B\right\}$ . Each bootstrap sample $D_{b}$ is used to build a decision tree. At each node, a subset of features is randomly selected for the splitting strategy, and the best feature within this subset is chosen for the split. If there are p total features, typically m features are selected, $m\approx \sqrt{p}$ . For regression problems, the final prediction is the average of all predictions from each tree: $\hat{y}=\frac{1}{B}\sum_{b=1}^{B}T_{b}\left(x\right)$ .

##### 4.2.1.5 SHAP interpretation of machine learning model

The SHAP (Shapley Additive Explanations) value was initially proposed to address the problem of reward distribution in cooperative game theory. In machine learning, the model’s prediction result can be seen as the outcome of the “cooperation” of all features. The SHAP value assigns a contribution value to each feature to explain its importance in the model output.

The process of calculating the SHAP value for a specific feature $X_{i}$ is as follows:

1. Perform weighting for all possible feature subsets S , where S does not contain the feature $X_{i}$ .
2. Calculate the difference in model output between the model $f\left(S\right)$ before adding the feature $X_{i}$ and the model $f\left(S\cup \left\{i\right\}\right)$ after adding the feature $X_{i}$ .
3. Calculate the contribution value $\phi_{i}$ for feature $X_{i}$ by averaging all these differences with weights.

As shown in Equation 5:

$$\phi_{i}=\sum_{S\subseteq N\left\{i\right\}}\frac{\left|S\right|!\left(\left|N\right|-\left|S\right|-1\right)!}{\left|N\right|!}\left[f\left(S\cup \left\{i\right\}\right)-f\left(S\right)\right]$$ (5)

where $\phi_{i}$ is the SHAP value for feature $X_{i}$ , S is a subset of features that does not include $X_{i}$ , N is the set of all features, $f\left(S\right)$ is the model output prediction for the feature subset S , and $\left|S\right|$ is the number of features in subset S .

**FIGURE 3:**
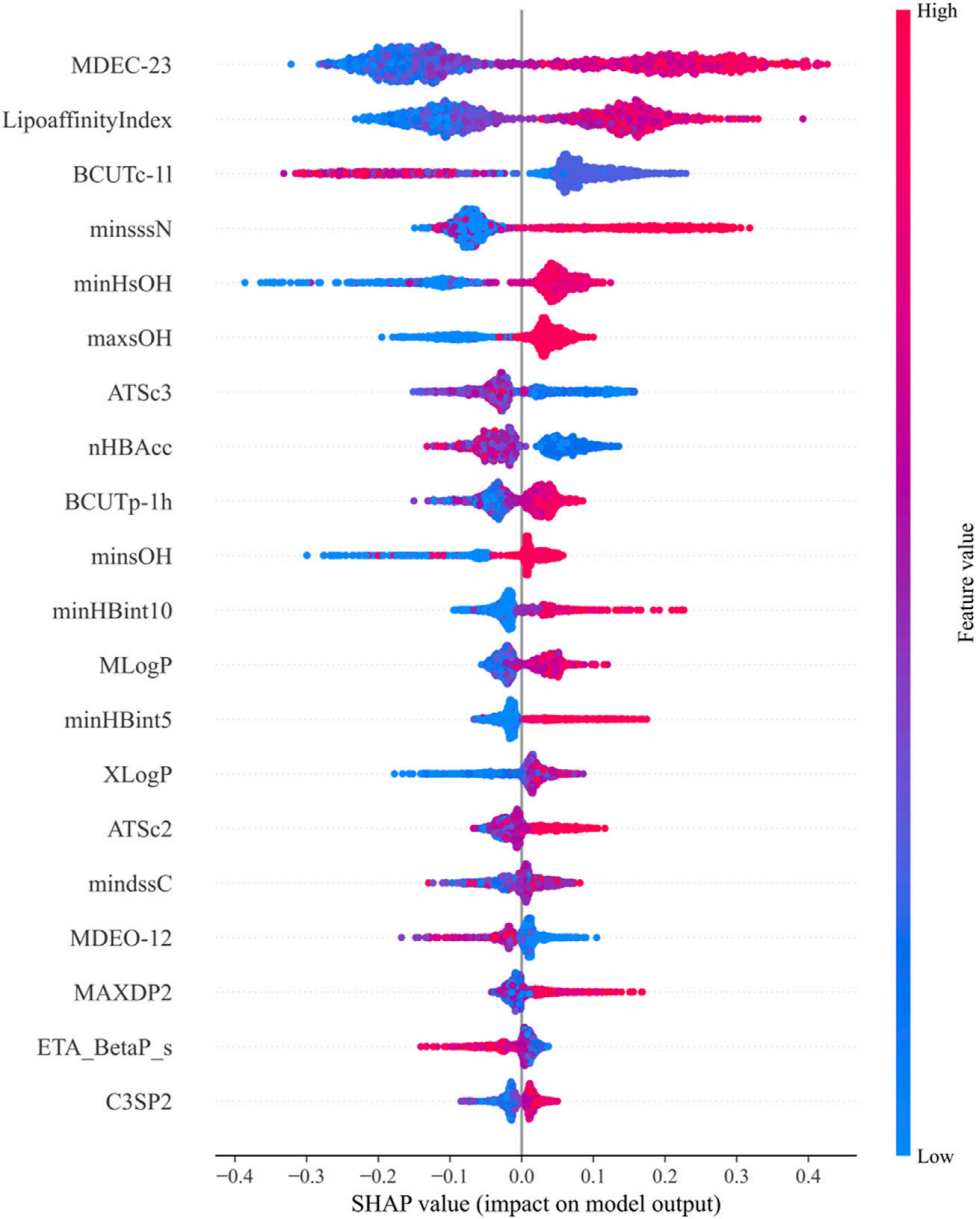
Top 20 molecular descriptors with the highest SHAP values based on the random forest model.

SHAP values are used to explain the contribution of features in machine learning models, assessing the specific impact of each feature on the model’s predictions. Through the above calculations, the top 20 molecular descriptors with the highest SHAP values were selected, representing the 20 descriptors with the most significant impact on biological activity, as shown in Figure 3. Each violin plot in the figure represents the SHAP value distribution for each molecular descriptor, with the SHAP value reflecting the extent to which the descriptor influences the model output.

In Figure 3:

1. The SHAP values of each molecular descriptor are mapped to dots of different colors, with the color bar on the right indicating the magnitude of the feature values. Blue represents low feature values, while red represents high feature values.
2. The horizontal axis represents the magnitude of SHAP values. The larger the SHAP value, the greater the positive contribution of the feature to the model’s prediction. Conversely, smaller SHAP values indicate a smaller contribution.
3. The shape of the violin plot shows the distribution of SHAP values at different feature values. A wider distribution indicates greater variation in the feature’s influence on the model output across different values.

The final selected molecular descriptors are shown in Table 2.

**TABLE 2:** The 20 molecular descriptors.

No.	Molecular descriptor
1	LipoaffinityIndex
2	BCUTc-1l
3	minsssN
4	minHsOH
5	maxsOH
6	ATSc3
7	nHBAcc
8	BCUTp-1h
9	minsOH
10	minHBint10
11	MEDC-23
12	MLogP
13	minHBint5
14	XLogP
15	ATSc2
16	mindssC
17	MDEO-12
18	MAXDP2
19	ETA_BetaP_s
20	C3SP2

### 4.3 Construction of biological activity prediction model

The feature variables selected are the 20 molecular descriptors shown in Table 2, with the data divided into training, testing, and validation sets in an 8:1:1 ratio.

1. Regression Model Selection: Ten common machine learning regression models were used, including Linear Regression, Ridge, Lasso, ElasticNet, RandomForest, LightGBM, XGBoost, Gradient Boosting Decision Tree (GBDT), SVM, and Decision Tree.
2. Multi-Model Fusion: To improve the predictive performance of the model, we experimented with three fusion strategies on the three best-performing models (LightGBM, RandomForest, and XGBoost), including simple averaging, weighted averaging, and stacking fusion. Stacking fusion yielded the best results.
3. Prediction Results: The optimal model was used to predict the pIC50 values for 50 test set compounds, which were then converted into IC50 values.

#### 4.3.1 Regression prediction model

##### 4.3.1.1 Linear regression

The linear regression model is a type of model that attempts to find the best linear relationship to describe the relationship between the target variable y and input features X . As shown in Equation 6:

y=Xβ+ϵ (6)

Where, X is the feature matrix, and β is the regression coefficient, and ϵ represents the error terms.

##### 4.3.1.2 Ridge regression

Ridge regression is an improved form of linear regression that incorporates an *L*_*2*_ regularization term into the regression model to reduce model complexity. As shown in Equation 7:

$$\hat{\beta }=\arg\min_{\beta }\left\{∥y-X\beta {∥}^{2}+\lambda ∥\beta {∥}^{2}\right\}$$ (7)

Where, λ is the regularization parameter.

##### 4.3.1.3 Lasso regression

Lasso regression introduces an L1 regularization term into the regression model, which can cause some regression coefficients to become zero. As shown in Equation 8:

$$\hat{\beta }=\arg\min_{\beta }\left\{∥y-X\beta {∥}^{2}+\lambda ∥\beta {∥}_{1}\right\}$$ (8)

Where λ is the regularization parameter.

##### 4.3.1.4 Elastic net

Elastic Net combines the advantages of Ridge Regression and Lasso Regression. As shown in Equation 9:

$$\hat{\beta }=\arg\underset{\beta }{\;\min}\left\{∥y-X\beta {∥}^{2}+\lambda_{1}∥\beta {∥}_{1}+\lambda_{2}∥\beta {∥}^{2}\right\}$$ (9)

Where $\lambda_{1}$ and $\lambda_{2}$ are the regularization parameters.

##### 4.3.1.5 XGBoost

XGBoost is an implementation of gradient boosting decision trees that provides optimized computational performance and memory usage. It accomplishes regression and classification tasks by incrementally enhancing the tree models. XGBoost employs regularization to prevent overfitting, as shown in Equation 10:

$$F\left(x\right)=\sum_{k=1}^{K}\alpha_{k}h_{k}\left(x\right)$$ (10)

Where $h_{k}\left(x\right)$ is the K -th tree, and $\alpha_{k}$ is its weight.

##### 4.3.1.6 LightGBM

LightGBM is an efficient implementation of gradient boosting decision trees that uses a histogram-based method to accelerate the training process and supports efficient handling of categorical features. It builds multiple trees incrementally, with each tree being optimized on the basis of gradient boosting. The model form is similar to that of XGBoost.

##### 4.3.1.7 Gradient boosting decision tree (GBDT)

GBDT is an ensemble learning method that builds multiple decision trees incrementally, with each tree attempting to correct the errors of the previous one to make predictions. The final prediction of the model is the weighted sum of all the decision tree predictions.

##### 4.3.1.8 Support vector machine (SVM)

SVM is a model for classification and regression that separates different categories of data points by finding the optimal hyperplane. As shown in Equation 11:

$$f\left(x\right)=\text{sgn}\left(w^{T}x+b\right)$$ (11)

Where w is the weight vector, and b is the bias term.

##### 4.3.1.9 Decision tree

Decision Tree is a tree-structured model that performs classification or regression by making conditional judgments on features. Each internal node represents a test on a feature, and each leaf node represents a class or value. As shown in Equation 12:

$$f\left(x\right)=leaf_{class}$$ (12)

Where x is the feature vector, and $f\left(x\right)$ is the predicted class.

#### 4.3.2 Model evaluation criteria

To measure the goodness of fit of the model, we used Mean Squared Error (MSE), Root Mean Squared Error (RMSE), Mean Absolute Error (MAE), Mean Absolute Percentage Error (MAPE), and R-Squared ( $R^{2}$ ) to evaluate the model.The calculation formula is shown in Table 3:

**TABLE 3:** Model evaluation metrics and their calculation formulas.

Evaluation metrics	Calculation formulas
MSE	$MSE=\frac{1}{m}\sum_{i=1}^{m}{\left(y_{i}-{\hat{y}}_{i}\right)}^{2}$
RMSE	$RMSE=\sqrt{\frac{1}{m}\sum_{i=1}^{m}{\left(y_{i}-{\hat{y}}_{i}\right)}^{2}}$
MAE	$MAE=\frac{1}{m}\sum_{i=1}\left|y_{i}-{\hat{y}}_{i}\right|$
MAPE	$MAPE=\frac{100\%}{n}\sum_{i=1}^{n}\left|\frac{{{\hat{y}}_{i-}y}_{i}}{y_{i}}\right|$
$R^{2}$	$R^{2}=1-\frac{\sum_{i=1}{\left({{\hat{y}}_{i-}y}_{i}\right)}^{2}}{\sum_{i=1}{\left({\bar{y}}_{i}-y_{i}\right)}^{2}}$

In Table 3, $y_{i}$ and ${\hat{y}}_{i}$ represent the actual and predicted values on the test set, respectively. The smaller the values of MSE, RMSE, MAE, and MAPE, the higher the predictive accuracy of the model. The closer the $R^{2}$ value is to 1, the better the model’s fit.

#### 4.3.3 Model solving

**FIGURE 4:**
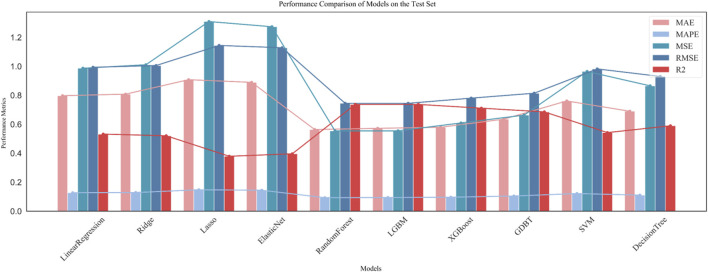
Comparison of ten regression models.

The feature variables selected are the 20 molecular descriptors listed in Table 2, which are divided into training, testing, and validation sets in an 8:1:1 ratio. Initially, ten different machine learning models were used for regression prediction. The predictive performance of these regression models is illustrated in Figure 4.

**FIGURE 5:**
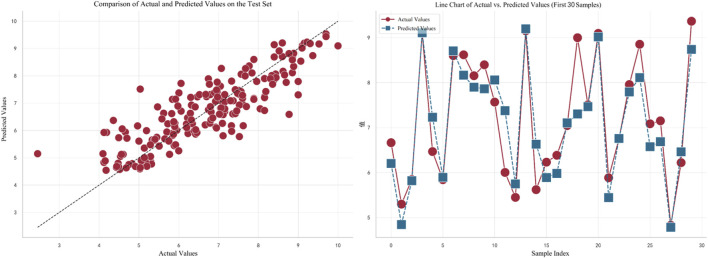
Prediction performance of the stacking model.

As can be seen, the three models with the highest $R^{2}$ values are LightGBM, RandomForest, and XGBoost, with values of 0.737, 0.736, and 0.711, respectively. To enhance the prediction accuracy, we experimented with multi-model fusion predictions. We selected the three models with the highest $R^{2}$ values and tried three types of fusion strategies: simple average fusion, weighted fusion (5:3:2), and stacking fusion, to improve the predictive performance of the models. The stacking fusion model, which showed the best predictive effect, achieved an $R^{2}$ value of 0.743. The predictive performance of the stacking model is depicted in Figure 5, and the final results were populated in “ERα_activity_test.csv.”

In Figure 5, the left plot displays a comparison between the actual values (on the horizontal axis) and predicted values (on the vertical axis) for the test set. Each red dot represents the corresponding actual and predicted value for a test sample, with the dashed line indicating a perfect prediction. It can be observed that the overall trend of the predictions is quite close to the perfect prediction line. The right plot shows a line chart of the actual values versus predicted values for the first 30 samples. Red dots represent actual values, and blue squares represent predicted values. The dashed line connecting these points illustrates the variation trend between the actual and predicted values for each sample. By observing this line, it can be concluded that the model fits the data well in most cases.

### 4.4 Classification prediction of ADMET properties

1. Recursive Feature Elimination (RFE): Using RandomForest as the base model, the Recursive Feature Elimination method was applied to select features for ADMET properties, selecting 25 most representative molecular descriptors for each ADMET attribute.
2. Classification Model Selection: Eleven classification models were used, including Logistic Regression, Naive Bayes, LDA, Decision Tree, RandomForest, AdaBoost, GradientBoosting, SVM, MLP, XGBoost, and LightGBM, to predict the ADMET properties of compounds.
3. Classification Performance Evaluation: Model performance was evaluated using metrics such as F1 score and ROC curve, and the best model was selected for each ADMET property. The best classification models for different ADMET properties were LightGBM (Caco-2), XGBoost (CYP3A4 and hERG), Naive Bayes (HOB), and XGBoost (MN).
4. ADMET Property Prediction: The selected best models were used to predict the ADMET properties of 50 compounds.

#### 4.4.1 Recursive feature elimination (RFE)

RFE is an algorithm used for feature selection. Its core idea is to recursively train a model and eliminate the least important feature after each training cycle based on the importance scores assigned to features. Assuming a dataset contains nnn features, RFE can be used to select the optimal subset of features.

#### 4.4.2 Classification prediction models

##### 4.4.2.1 Logistic Regression

Logistic regression is a linear model used for binary classification problems. It maps the predicted values to probabilities by applying the sigmoid function to a linear combination of features. As shown in Equation 13:

$$P\left(y\left.=1\right|X\right)=\frac{1}{1+e^{-\left(\beta_{0}+\beta^{T}X\right)}}$$ (13)

Where X is the feature vector, β is the regression coefficient vector, and $\beta_{0}$ is the bias term.

##### 4.4.2.2 Naive Bayes

The Naive Bayes classifier is a simple classifier based on Bayes’ theorem, assuming that features are independent of each other. As shown in Equation 14:

$$P\left(y|X\right)=\frac{P\left(y\right)\prod_{i=1}^{n}P\left(x_{i}|y\right)}{P\left(X\right)}$$ (14)

Where, y is the class label, X is the feature vector, and $x_{i}$ is the i -th feature.

##### 4.4.2.3 Linear discriminant analysis (LDA)

LDA is a technique used for dimensionality reduction and classification. It seeks to find the projection direction that maximizes between-class scatter while minimizing within-class scatter. The objective is to find the optimal linear transformation by maximizing the ratio of between-class scatter to within-class scatter, As shown in Equation 15:

$$J\left(w\right)=\frac{w^{T}S_{B}w}{w^{T}S_{W}w}$$ (15)

Where $S_{B}$ is the between-class scatter matrix, $S_{W}$ is the within-class scatter matrix, and w is the projection vector.

##### 4.4.2.4 Adaptive boosting (AdaBoost)

AdaBoost is an ensemble learning method that iteratively trains a series of weak classifiers (e.g., decision stumps), with each classifier improving upon the previous one. The final classification result is a weighted vote of all weak classifiers. As shown in Equation 16:

$$f\left(x\right)=\sum_{m=1}^{M}\alpha_{m}h_{m}\left(x\right)$$ (16)

where $h_{m}\left(x\right)$ is the m -th weak classifier, and $\alpha_{m}$ is its weight.

##### 4.4.2.5 Gradient boosting

Gradient Boosting Trees is an ensemble learning method that builds decision trees sequentially, where each tree attempts to correct the errors of the previous trees. The model’s final prediction is the weighted sum of all decision trees’ predictions. As shown in Equation 17:

$$F\left(x\right)=F_{m-1}\left(x\right)+\eta \cdot h_{m}\left(x\right)$$ (17)

where $F_{m-1}\left(x\right)$ is the prediction from the first m−1 trees, $h_{m}\left(x\right)$ is the m -th tree, and η is the learning rate.

##### 4.4.2.6 MLP

A Multilayer Perceptron is a feedforward neural network consisting of an input layer, one or more hidden layers, and an output layer. Each layer comprises multiple neurons that perform nonlinear transformations through activation functions (such as ReLU, Sigmoid, etc.). As shown in Equation 18:

$$a^{\left(l\right)}=\sigma \left(W^{\left(l\right)}a^{\left(l-1\right)}+b^{\left(l\right)}\right)$$ (18)

Where $a^{\left(l\right)}$ is the activation vector of the l -th layer, $W^{\left(l\right)}$ is the weight matrix of the l -th layer, $b^{\left(l\right)}$ is the bias term, and σ is the activation function.

#### 4.4.3 Model evaluation metrics

To select the most effective models, this study utilizes the following classification algorithm evaluation metrics to assess the performance of each model. Let us define:

True Positives (tp): the number of samples correctly predicted as class 1 (predicted as 1 and actually being 1).

False Positives (fp): the number of samples incorrectly predicted as class 1 (predicted as 1 but actually being 0).

False Negatives (fn): the number of samples incorrectly predicted as class 0 (predicted as 0 but actually being 1).

True Negatives (tn): the number of samples correctly predicted as class 0 (predicted as 0 and actually being 0).

##### 4.4.3.1 F1 score

The F1 score is a weighted measure of precision and recall, defined as the harmonic mean of precision and recall. As shown in Equation 19:

$$F1=2\frac{\text{Precision}\times \text{Recall}}{\text{Precision}+\text{Recall}}$$ (19)

Where, $\text{Precision}=\frac{TP}{TP+FP}$ , $\text{Recall}=\frac{TP}{TP+FN}$ . In model evaluation, a higher F1 score indicates better performance.

##### 4.4.3.2 ROC curve

The ROC curve, also known as the Receiver Operating Characteristic curve, is a graphical tool used in binary classification problems. In this context, each point on the ROC curve represents a specific threshold. The classifier assigns a score to each sample; if the score exceeds the threshold, the sample is classified as a positive instance; if it is below the threshold, it is classified as a negative instance. The closer the ROC curve is to the upper-left corner of the plot, the better the classification performance of the model.

#### 4.4.4 Model solving

##### 4.4.4.1 Data preprocessing

Initially, using the molecular descriptors remaining after removing single-value variables from Problem 1, the Recursive Feature Elimination (RFE) algorithm was used to select 25 feature variables corresponding to each ADMET property. The specific feature selections for each property are shown in Tables 4–8.

**TABLE 4:** Selected features for Caco-2.

No.	Molecular descriptor
1	BCUTc-1h
2	SP-1
3	SP-2
4	ECCEN
5	SHBd
6	SHother
7	SsCH3
8	SaaO
9	minHBa
10	minwHBa
11	minaaO
12	maxaaO
13	ETA_Alpha
14	ETA_Beta_s
15	ETA_Eta_R_L
16	FMF
17	MDEC-23
18	MLFER_S
19	MLFER_L
20	TopoPSA
21	MW
22	WTPT-1
23	WTPT-3
24	WTPT-4
25	WPATH

**TABLE 5:** Selected features for CY3A4.

No.	Molecular descriptor
1	ATSc1
2	bpol
3	VCH-6
4	SP-4
5	SP-7
6	VP-2
7	VP-4
8	VP-7
9	SHaaCH
10	ETA_dEpsilon_D
11	ETA_Eta
12	WTPT-1
13	Zagreb
14	ATSc2
15	SCH-7
16	SP-3
17	SP-5
18	VP-1
19	VP-3
20	VP-5
21	SHBd
22	minHBa
23	ETA_Beta_s
24	ETA_Eta_L
25	WTPT-3

**TABLE 6:** Selected Features for hERG.

No.	Molecular descriptor
1	ATSc2
2	bpol
3	VP-0
4	CrippenMR
5	SHBint8
6	SsOH
7	maxHBd
8	maxaaCH
9	LipoaffinityIndex
10	ETA_EtaP_F
11	Kier2
12	McGowan_Volume
13	WPATH
14	BCUTc-1l
15	SP-1
16	VP-1
17	ECCEN
18	SHother
19	minaasC
20	maxHsOH
21	hmin
22	ETA_EtaP
23	Kier1
24	Kier3
25	MDEO-11

**TABLE 7:** Selected features for HOB.

No.	Molecular descriptor
1	ATSc2
2	BCUTp-1l
3	VP-3
4	VP-6
5	SHsOH
6	SdO
7	minsOH
8	maxsOH
9	hmin
10	ETA_BetaP_s
11	ETA_EtaP_F_L
12	MLFER_A
13	WTPT-4
14	BCUTc-1l
15	SC-5
16	VP-5
17	VP-7
18	SsOH
19	minHBa
20	maxHsOH
21	maxdO
22	ETA_Shape_P
23	ETA_EtaP_L
24	Kier3
25	MLFER_BO

**TABLE 8:** Selected features for MN.

No.	Molecular descriptor
1	nN
2	VPC-5
3	SssCH2
4	minHBa
5	maxsCH3
6	ETA Epsilon 1
7	ETA dEpsilon A
8	ETA BetaP
9	ETA EtaP B RC
10	nHBAcc Lipinski
11	MLFER E
12	WTPT-3
13	WTPT-5
14	SCH-7
15	nssCH2
16	SssO
17	mindssC
18	maxsssCH
19	ETA Epsilon 4
20	ETA dEpsilon C
21	ETA BetaP s
22	FMF
23	MLFER S
24	TopoPSA
25	WTPT-4

##### 4.4.4.2 Results of the model in ADMET property prediction

**FIGURE 6:**
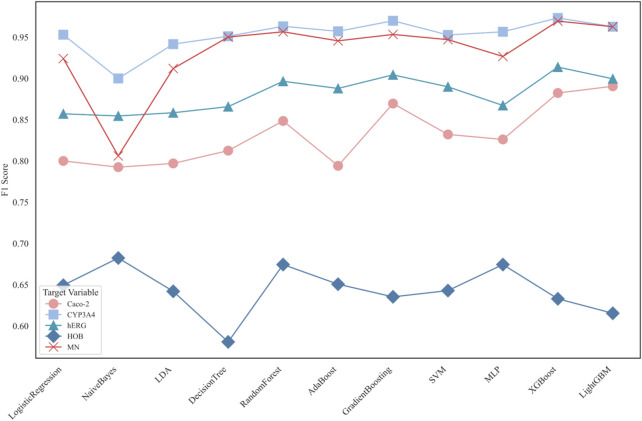
Comparison of F1 scores for 11 classification models in ADMET property prediction.

Subsequently, eleven machine learning models were used to classify the five ADMET features individually. The F1 scores of each model’s prediction results are shown in Figure 6.

Figure 6 displays the F1 scores of different classification models for five distinct ADMET properties: Caco-2, CYP3A4, hERG, HOB, and MN. The performance of 11 classification models is compared using line charts. Each target variable is represented by different symbols to distinguish their performance in predictions.

Application Results of Different Models in ADMET Property Prediction:

In the confusion matrix of the following set of figures, the symbols represent the following meanings.

1. True 0: Samples where the actual value is 0 (poor intestinal absorption).
2. True 1: Samples where the actual value is 1 (good intestinal absorption).
3. Predicted 0: Samples predicted as 0 by the model.
4. Predicted 1: Samples predicted as 1 by the model.

**FIGURE 7:**
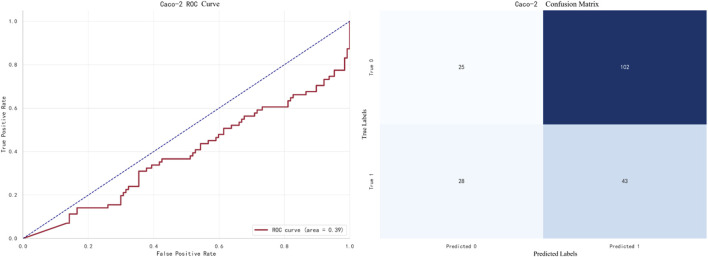
LightGBM prediction for Caco-2.

The best-performing model for Caco-2 prediction is LightGBM, with an F1 score of **0.8905**. The ROC curve and confusion matrix are shown in Figure 7.

**FIGURE 8:**
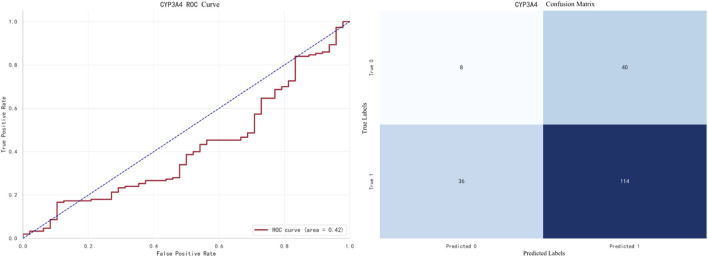
XGBoost prediction for CYP3A4.

The best-performing model for CYP3A4 prediction is XGBoost, with an F1 score of **0.9733**. The ROC curve and confusion matrix are shown in Figure 8.

**FIGURE 9:**
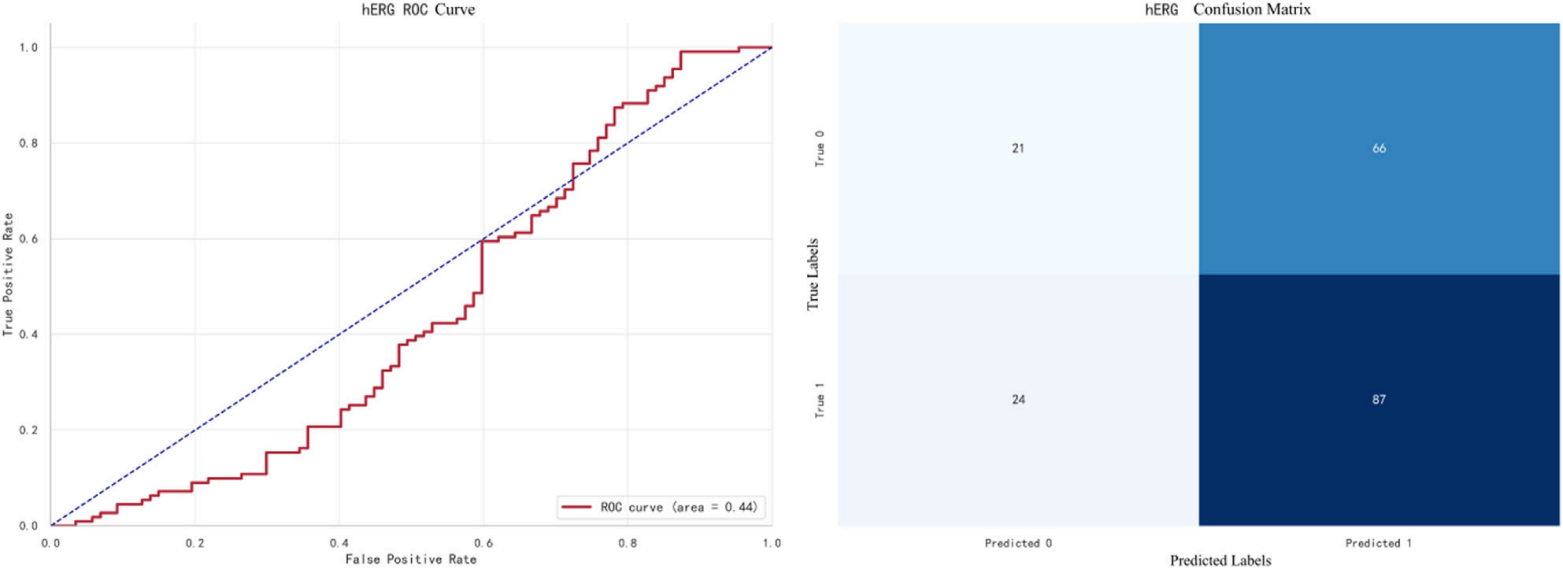
XGBoost Prediction for hERG.

The best-performing model for hERG prediction is XGBoost, with an F1 score of **0.9138**. The ROC curve and confusion matrix are shown in Figure 9.

**FIGURE 10:**
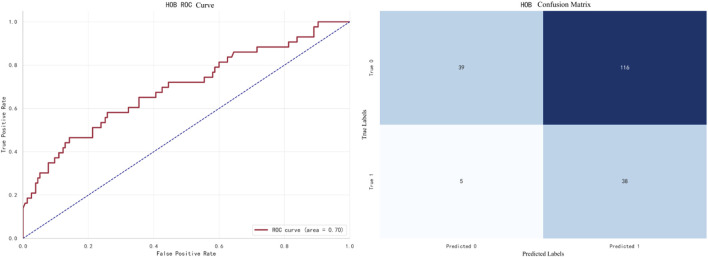
NaiveBayes prediction for HOB.

The best-performing model for HOB prediction is Naive Bayes, with an F1 score of **0.6824**. The ROC curve and confusion matrix are shown in Figure 10.

**FIGURE 11:**
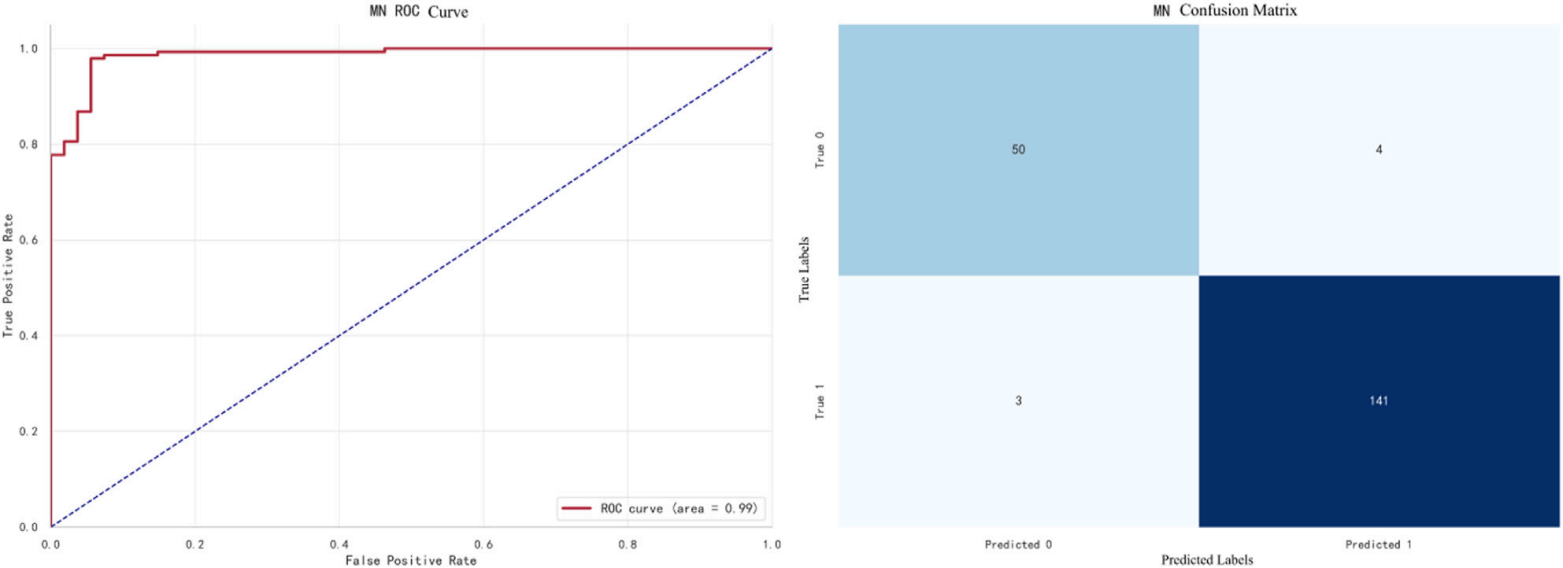
XGBoost prediction for MN.

The best-performing model for MN prediction is XGBoost, with an F1 score of **0.9695**. The ROC curve and confusion matrix are shown in Figure 11.In this figure, the AUC (Area Under the Curve) of the ROC curve is 0.99, indicating that the model performs exceptionally well in the MN prediction task.

Finally, we used the best-performing models to predict the ADMET properties of 50 compounds, and the final results were entered into “ADMET_test.csv.”

### 4.5 Multi-objective optimization

1. Single-Objective Optimization: Establish a single-objective optimization model with the goal of enhancing the biological activity (pIC50 value) of the compounds while ensuring that at least three ADMET properties perform well.
2. Particle Swarm Optimization (PSO): Utilize the PSO algorithm to globally optimize 106 important features, recording the optimal solution in each iteration, and ultimately finding the value range that provides the best performance in both biological activity and ADMET properties.
3. Final Results: Apply the optimized compound features to 50 test compounds, outputting their optimal predicted values.

#### 4.5.1 Constrained optimization

A constrained optimization problem (COP) involves optimizing an objective function under specific constraints. In this case, we can establish a constrained optimization model to solve the problem.

##### 4.5.1.1 Decision variables

In the model established for this problem, there are a total of 106 molecular descriptors that affect both the biological activity and ADMET properties of the compounds. This includes 20 molecular descriptors affecting biological activity identified in the first question, and 25 descriptors affecting each ADMET property identified in the third question, with 39 of these descriptors being duplicates.

The decision variable x is denoted as: $x={\left[x_{1},x_{2},\cdots ,x_{106}\right]}^{T}$

##### 4.5.1.2 Objective function and constraints

As shown in Equation 20: Objective Function:

$$\begin{array}{rr} & \min{\;F}_{PIC50}\left(x\right)\\ & s.t.\text{ Reward}\left(g_{i}\left(x\right)\right)\geq 3\\ & \;x_{i}^{\left(L\right)}\leq x_{i}\leq x_{i}^{\left(U\right)},i=1,2,\cdots ,p\\ & \;x\in R^{n} \end{array}$$ (20)

Where: $F\left(x\right)$ represents the biological activity prediction function for the compound. $g_{i}\left(x\right)$ , i=1,2,3,4,5 represent the classification models for the ADMET properties affecting the compound.

The reward function $\text{Reward}\left(g_{i}\right)$ is given by: $\text{Reward}\left(g_{i}\right)=\left(g_{i}\right)=g_{1}+g_{2}+\left(1-g_{3}\right)+g_{4}+\left(1-g_{5}\right)$ . Here, $g_{1}$ represents the Caco-2 classification model, $g_{2}$ represents the CYP3A4 classification model, $g_{3}$ represents the hERG classification model, $g_{4}$ represents the HOB classification model, $g_{5}$ represents the MN classification model.Assuming that the optimal combination is achieved when Caco-2 is set to 1, CYP3A4 is set to 1, hERG is set to 0, HOB is set to 1, and MN is set to 0, the reward function becomes Reward = 5 under these conditions.

The requirement is met as long as the Reward function value is greater than or equal to 3.

#### 4.5.2 Particle swarm optimization algorithm for finding optimal solutions

Particle Swarm Optimization (PSO), a concept inspired by the simulation of birds foragingBy designing particles to simulate birds, which represent feasible solutions to optimization problems, each particle possesses three attributes—velocity, position, and fitness value. Each particle independently searches for the best solution in the search space, known as the personal best, and shares it with all particles in the swarm. The best of these personal bests is considered the current global best solution for the entire swarm. All particles then adjust their positions based on this global best and their own personal bests until a globally optimal solution that meets the criteria is found.

Assume a swarm of m particles in a D -dimensional target search space. The properties of the i -th particle at time t consist of two vectors:

1. Velocity: $v_{i}^{t}=\left(v_{i1}^{t},v_{i2}^{t},\cdots ,v_{id}^{t}\right)$ , $v_{id}^{t}\in \left[v_{\min\;},v_{\max\;}\right]$ . Where $v_{\min}$ and $v_{\max}$ represent the minimum and maximum components of the velocity, respectively.
2. Position: $x_{i}^{t}=\left(x_{i1}^{t},x_{i2}^{t},\cdots ,x_{id}^{t}\right)$ , $x_{id}^{t}\in \left[l_{d},u_{d}\right]$ . Where $l_{d}$ and $u_{d}$ are the lower and upper bounds of each particle’s search space components.

In each iteration, two optimal positions are recorded.

1. Individual optimal position: $p_{i}^{t}=\left(p_{i1}^{t},p_{i2}^{t},\cdots ,p_{id}^{t}\right)$ ;
2. Global optimal position: $p_{g}^{t}=\left(p_{g1}^{t},p_{g2}^{t},\cdots ,p_{gd}^{t}\right)$ ; where $1\leq i\leq M,1\leq \dot{d}\leq D$ .

According to the above theory, the velocity and position of the particle are updated at time t+1 and the formulas are shown in Equations 21, 22:

$$v_{i}^{t+1}=v_{i}^{t}+c_{1}r_{1}\left(p_{i}^{t}-x_{i}^{t}\right)+c_{2}r_{2}\left(p_{g}^{t}-x_{i}^{t}\right)$$ (21)

$$x_{i}^{t+1}=x_{i}^{t}+v_{i}^{t+1}$$ (22)

Here, $r_{1}$ and $r_{2}$ are random numbers in the range (0,1), and $c_{1}$ and $c_{2}$ are learning factors.

#### 4.5.3 Model solving

The selected feature variables consist of the 20 variables most highly correlated with biological activity, identified in the first question, and the top 25 variables most highly correlated with each of the five ADMET properties, identified in the third question. There are 39 duplicate variables, making a total of 106 feature variables.

**FIGURE 12:**
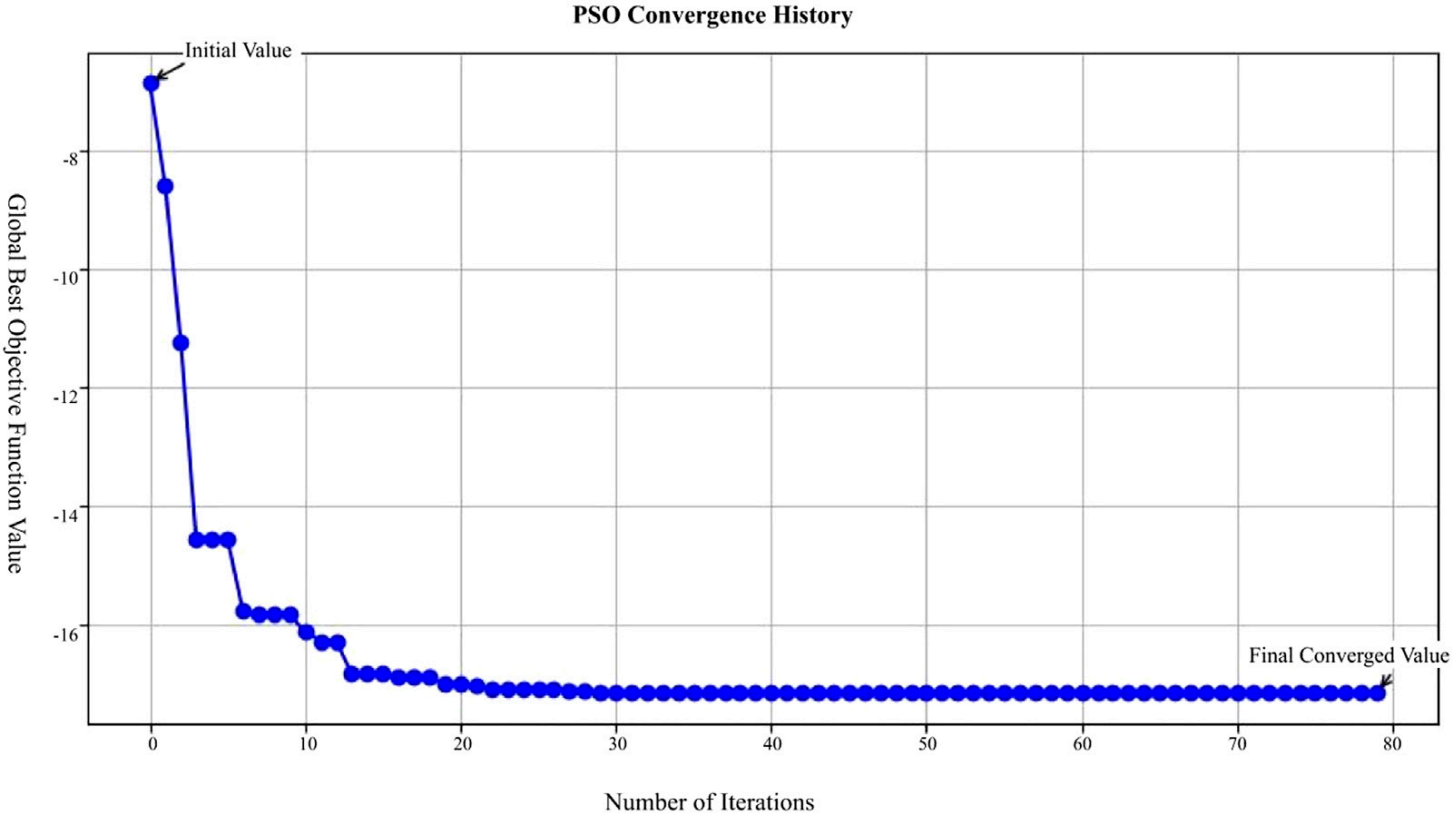
Optimal values obtained using the particle swarm optimization algorithm.

In our Particle Swarm Optimization approach, after various trials and adjustments, we determined the optimal parameters: the inertia weight w = 0.8, cognitive coefficient $c_{1}=0.5$ , and social coefficient $c_{2}=0.5$ . The convergence process is illustrated in Figure 12.

In Figure 12, the X-axis represents the number of iterations in the Particle Swarm Optimization (PSO) process, ranging from 0 to 80 iterations; the Y-axis represents the global best objective function value after each iteration; the blue curve in the figure shows the trend of the objective function value, starting from the initial value and decreasing rapidly with each iteration, eventually stabilizing and approaching the final converged value.

This figure demonstrates that the PSO algorithm converges rapidly after multiple iterations, with the objective function value gradually decreasing from an initially high value and eventually stabilizing, indicating that the optimization process effectively finds a solution.

The optimal value ranges for some molecular descriptors are shown in Table 9. The complete results are available in the attached document “results.csv.”

**TABLE 9:** Optimal value ranges for molecular descriptors.

Molecular descriptors	Optimal value ranges
ATSc1	(0.03, 1.89)
ATSc3	(−0.37, −0.16)
BCUTc-1l	(−0.32, −0.19)
ATSc2	(−2.38, −1.00)
BCUTc-1h	(0.07, 0.33)
BCUTp-1h	(7.97, 16.75)
BCUTp-1l	(3.01, 7.00)
CrippenMR	(56.15, 400.61)
C3SP2	(0.00, 9.30)
ECCEN	(196.00, 1294.89)

## 5 Results

This study proposes a machine learning-based optimization model for anti-breast cancer candidate drugs, which has achieved significant results in enhancing the biological activity of compounds and optimizing their ADMET (absorption, distribution, metabolism, excretion, toxicity) properties. After feature selection from 1,974 compounds, 20 molecular descriptors highly correlated with biological activity were retained. The QSAR (Quantitative Structure-Activity Relationship) model built upon these descriptors demonstrates high predictive accuracy. The results of the conducted experiments are presented below, highlighting the performance of the various models used in this study. A comparison of performance metrics for different regression and classification models is shown, with models being evaluated based on their ability to predict biological activity (pIC50 values) and ADMET properties. The metrics include R^2^ for regression tasks, and F1 score and accuracy for classification tasks. As shown in Table 10, the stacking ensemble model performed the best in predicting biological activity, achieving an R^2^ value of 0.743. For ADMET property prediction, models such as XGBoost and LightGBM achieved the highest F1 scores for specific properties, detailed further in Table 10.

**TABLE 10:** Comparison of model performance.

Model	Task	R^2^/F1 score	Accuracy/AUC
LightGBM	Biological Activity	0.737	—
RandomForest	Biological Activity	0.736	—
XGBoost	Biological Activity	0.711	—
Stacking Ensemble	Biological Activity	**0.743**	—
LightGBM	Caco-2 Prediction	—	**0.8905**
XGBoost	CYP3A4 Prediction	—	**0.9733**
XGBoost	hERG Prediction	—	**0.9138**
Naive Bayes	HOB Prediction	—	**0.6824**
XGBoost	MN Prediction	—	**0.9695**

The stacking ensemble model achieved an excellent R^2^ value of 0.743 for predicting biological activity. In terms of ADMET property prediction, XGBoost performed best for predicting CYP3A4 and MN, while Naive Bayes demonstrated strong performance in predicting HOB. By applying the Particle Swarm Optimization (PSO) algorithm, effective multi-objective optimization was performed for both biological activity and ADMET properties. The optimized compounds met the pre-defined combination of ADMET properties and exhibited good biological activity. Ultimately, the 50 optimized test compounds achieved ideal predictive results for both biological activity and ADMET properties, validating the effectiveness and practicality of this model in the development of anti-breast cancer drugs.

## 6 Discussion

This study proposes a machine learning-based optimization model for anti-breast cancer candidate drugs, which has made significant progress in enhancing the biological activity of candidate compounds and optimizing their ADMET properties. However, there are still several potential directions for future research and practical applications.

### 6.1 Future research directions

With the continuous development of drug discovery and optimization, this study opens several potential avenues for future progress:

#### 6.1.1 Incorporating more data

While this study primarily relies on molecular descriptors and biological activity data, future research could consider incorporating more diverse datasets, such as gene expression profiles, protein-ligand interactions, and *in vivo* pharmacokinetic data. These additional data could improve the robustness of the model and enhance the generalizability of predictions.

#### 6.1.2 Exploring other optimization algorithms

Although Particle Swarm Optimization (PSO) has shown effective results in multi-objective optimization, exploring other optimization algorithms such as Genetic Algorithms (GA), Differential Evolution (DE), or multi-objective versions of Reinforcement Learning could potentially extend the model’s applicability to drug screening and optimization for other diseases.

#### 6.1.3 Applying the model to other cancer types

While this study focuses on breast cancer, the machine learning-based optimization approach can be extended to other types of cancer. Future research can incorporate biomarkers and therapeutic targets specific to different diseases and apply the model to various cancer targets, such as ovarian cancer, lung cancer, or prostate cancer. This would broaden the scope and applicability of the model, making it a valuable tool in the global fight against cancer.

### 6.2 Practical applications of the model

The model proposed in this study not only provides theoretical insights but also has great potential in the practical application of drug development and personalized medicine:

#### 6.2.1 Early drug discovery screening

The multi-objective optimization model can be applied in the early stages of drug discovery to screen large compound libraries. By predicting both biological activity and ADMET properties simultaneously, the model can help researchers identify promising lead compounds with favorable characteristics, reducing experimental screening time and costs. This can accelerate the identification of promising drug candidates, especially in cancer treatment.

#### 6.2.2 Personalized cancer therapy

In the context of precision medicine, the model can be used to optimize drugs based on individual patients’ genomic profiles and tumor characteristics. By predicting how specific compounds interact with a patient’s unique molecular features, this approach can contribute to the development of more effective and personalized treatment plans, ultimately improving patient outcomes and reducing side effects.

#### 6.2.3 Optimizing existing drugs

The model can also be applied to optimize existing anti-cancer drugs that are already in clinical use. By fine-tuning their biological activity and ADMET properties, the model can suggest modifications or derivatives of these drugs to overcome existing limitations such as drug resistance, toxicity, or poor bioavailability. This can enhance the therapeutic effectiveness of existing drugs and provide new treatment options for patients.

#### 6.2.4 Integration into drug discovery platforms

In industrial settings, the model can be integrated into drug discovery platforms as a valuable decision-support tool. Pharmaceutical companies can use the model to guide their drug development strategies, especially during the preclinical phase. The ability to predict the combined impact of biological activity and ADMET properties on the success of drug candidates will be a key asset in determining which compounds should proceed to further testing and clinical development.

## 7 Conclusion

This study proposes an optimization model for anti-breast cancer candidate drugs based on machine learning and particle swarm optimization, achieving significant results in enhancing the biological activity and ADMET properties of candidate compounds. Through grey relational analysis, Spearman correlation analysis, and SHAP value screening from the random forest model, 20 molecular descriptors most influential to biological activity were successfully selected. A multi-model fusion technique was applied to improve the accuracy of biological activity predictions. The use of efficient classification models in ADMET property prediction further ensures the superior pharmacokinetic performance of candidate drugs. The successful application of the particle swarm optimization algorithm in multi-objective optimization tasks demonstrates its potential in drug design.

The model proposed in this study provides a novel and efficient solution for the field of drug design and development, accelerating the development process of new anti-breast cancer drugs and offering theoretical foundations and technical support for future multi-objective drug optimization. Future research will focus on validation and optimization on large-scale datasets, integrating laboratory data to further improve the performance of machine learning models, thereby achieving a closed-loop development process from computational prediction to experimental validation.

## Data Availability

The original contributions presented in the study are included in the article/Supplementary Material, further inquiries can be directed to the corresponding author.
